# Morphometric Analysis of the Mandibular Condyle and Ramus on Panoramic Radiographs: A Cross-Sectional Study in Hail, Saudi Arabia

**DOI:** 10.7759/cureus.101844

**Published:** 2026-01-19

**Authors:** Yosef Alanazi, Eyad A Almagdawi, Rayan B Alanazi, Faisal Y Alharbi, Abdulaziz K Alduayyi, Ahmed S Altheban

**Affiliations:** 1 Oral and Maxillofacial Surgery, College of Dentistry, University of Hail, Hail, SAU; 2 College of Dentistry, Dental Research Center, University of Hail, Hail, SAU; 3 Radiology, Dental Research Center, University of Hail, Hail, SAU; 4 College of Nursing, Dental Research Centre, University of Hail, Hail, SAU

**Keywords:** condylar morphology, mandibular condyle, panoramic radiography, ramus height, sexual dimorphism

## Abstract

Introduction: The mandibular condyle and ramus exhibit considerable anatomical variation, with morphology influenced by growth, function, occlusion, and age-related remodeling. While cone-beam computed tomography (CBCT) provides high diagnostic accuracy, panoramic radiography remains widely used due to its accessibility and low radiation burden. This study aimed to assess radiographic condylar morphology, measure condylar and ramus dimensions, and examine associations with age, sex, and posterior dentition in adults in Hail, Saudi Arabia.

Methodology: A retrospective cross-sectional study analyzed 366 digital panoramic radiographs obtained between 2023 and 2024 at the University of Hail dental polyclinics. Condylar shape, condylar height and width, ramus height, total mandibular height, intercondylar distance, and posterior molar presence were recorded bilaterally. Measurements were performed in Sirona Dental Imaging Software (SIDEXIS XG) using standardized landmarks. Associations with sex and age were assessed using chi-square tests, with *P *< 0.05 considered significant.

Results: The round condylar morphology was the most common bilaterally - right side: 241 (75.8%) and left side: 262 (71.6%). Ramus height showed significant sexual dimorphism, with higher values in males (*n* = 93, 57.4%) compared with females (*n* = 91, 44.6%) in the 45-55 mm category. Age was significantly associated with condylar width, condylar height, ramus height, and total mandibular height across all groups. Posterior molar presence demonstrated a marked age-related decline. For example, the upper right first molar was present in 47 individuals (95.9%) aged 18-20 years but in only 11 individuals (50.0%) aged ≥60 years. Intercondylar distance showed no significant differences by sex or age.

Conclusions: Panoramic radiography reliably demonstrated condylar morphology and vertical mandibular dimensions in this population. Round condyles were the predominant morphology, and ramus height was the only parameter consistently exhibiting sexual dimorphism. Age strongly influenced mandibular vertical dimensions and molar presence. Panoramic radiographs remain valuable for population-level screening, though CBCT is necessary when detailed morphometric evaluation is required.

## Introduction

The temporomandibular joint (TMJ) is composed of several distinct anatomical elements, including the mandibular condyle, the mandibular fossa, the articular eminence of the temporal bone, and the soft-tissue structures of the articular disc with its associated attachments and joint space. The mandibular condyle is particularly important because it plays a central role in expressing and guiding mandibular growth [1]. Its capacity to translate enables the mandible to achieve a far greater maximal incisal opening than would be possible through rotational movement alone [2]. Anatomical variations within the TMJ can significantly influence arthroscopic accessibility and the complexity of surgical procedures [3]. Moreover, developmental differences, adaptive remodeling driven by growth demands, trauma, malocclusion, and other developmental disturbances may contribute to distinct morphological changes of the condyle [1]. TMJ disorders are multifactorial in etiology, may become chronic, and are known to significantly affect patients’ quality of life, underscoring the clinical importance of understanding TMJ morphology and its variations [4].

A previous investigation reported that anterior displacement of the temporomandibular disk is strongly associated with alterations in both the size and shape of the mandibular condyles [5]. In addition, the radiographic appearance of the condyle demonstrates substantial variability across different age groups and among individuals. Even within the same person, the right and left condyles may differ in appearance, reflecting natural intraindividual morphological variations [3]. Numerous studies have examined the morphology and dimensions of the TMJ, particularly the mandibular condyles, and consistently found that the oval configuration is the most predominant form in both genders [6-8]. Furthermore, evidence indicates that sexual dimorphism within the mandible is marked, especially in the ramus and condylar regions, where structural differences appear to be influenced by variations in masticatory forces [9]. Panoramic radiography has been widely recommended as an initial screening modality for patients with TMJ-related symptoms, as it remains an effective tool for detecting major bony alterations in condylar morphology [10].

Given the functional, anatomical, and clinical significance of the mandibular condyle and ramus, along with the considerable variation observed among individuals, the present study aims to assess the radiographic morphology of the mandibular condyles in adults using panoramic radiographs, evaluate condylar and ramus linear measurements, identify variations in condylar shape, and explore potential associations with demographic factors. Accordingly, the objective of this study was to evaluate mandibular condylar morphology and linear measurements on panoramic radiographs in adults and to examine their associations with sex, age, and posterior dentition status. The primary outcome of this study was to evaluate sexual dimorphism in mandibular linear measurements, particularly ramus height, while secondary outcomes included assessment of condylar morphology and its associations with age and posterior dentition status.

## Materials and methods

Study setting

A cross-sectional retrospective study conducted in Hail, Saudi Arabia, aimed to assess the distribution and radiographic characteristics of mandibular condylar morphology among adults. Ethical approval was obtained from the Institutional Review Board (IRB) at the University of Hail. Radiographic records were retrieved from the electronic database of the University of Hail dental polyclinics for the period 2023-2024.

A sampling frame was constructed from all adult patients (≥18 years) who underwent routine panoramic radiography during the study period. After applying inclusion and exclusion criteria, eligible radiographs were assigned unique identifiers, and a simple random sample was selected for analysis. The minimum required sample size was calculated using a single-proportion formula, assuming a 70% prevalence of predominant round/oval condylar morphology based on prior literature, a 95% confidence level, and a 5% margin of error. The estimated minimum sample size was 323 radiographs. The final sample included 366 panoramic radiographs, exceeding the calculated requirement and ensuring adequate statistical power for subgroup and gender-based comparisons.

The study focused on evaluating potential factors that may influence condylar morphology, including linear measurements of the condyle and ramus, condylar shape variation, and associated dental status. The dental polyclinics at the University of Hail serve a diverse population, allowing for the collection of comprehensive and representative radiographic data.

Systematic evidence indicates that routine orthodontic and temporomandibular disorder assessment relies primarily on clinical evaluation, with conventional imaging used as adjunctive screening and advanced imaging reserved for specific indications [11]. Therefore, panoramic radiography was selected as it's a first-line imaging modality due to its wide availability, low radiation dose, and suitability for population-based screening of osseous mandibular and TMJ structures.

Data collection

Patient data were securely stored and fully anonymized to ensure confidentiality. The inclusion criteria were individuals aged 18 years and above, the presence of a clear and non-distorted panoramic radiograph, and the absence of systemic diseases that may influence bone development. Any radiograph that did not meet these criteria was excluded. Data were collected using a structured questionnaire designed by the researchers. 

All panoramic radiographs were obtained using a Sirona digital panoramic imaging system (Sirona Orthophos series; Dentsply Sirona, Bensheim, Germany). The device operates with standardized panoramic acquisition parameters ranging from 60 to 90 kVp and 6 to 15 mA, with an average exposure time of 14 to 16 seconds. The software system used for sample selection and image analysis was Sirona Dental System GmbH (SIDEXIS XG, version 2.63), operating on a Windows platform.

Measurements were performed using the SIDEXIS XG linear measurement tool with images viewed at standardized display settings (zoom level held constant during measurement, and brightness/contrast adjusted only when required to visualize landmarks).

**Figure 1:**
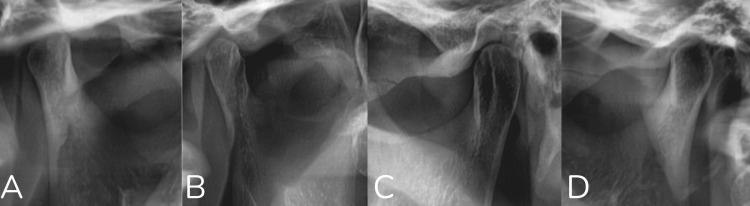
Radiographic classification of mandibular condylar morphology on panoramic radiographs. (A) Flat condyle, characterized by a flattened superior surface. (B) Angled condyle, showing a pointed or angular superior contour. (C) Round condyle, with a smooth, convex, rounded superior outline. (D) Convex condyle, demonstrating a prominent outward curvature without angularity.

The questionnaire addressed multiple aspects, including demographic information and detailed radiographic assessments for both sides of the mandible. Demographic data included age and sex. The extracted data included condylar shape, categorized as flat, convex, round, or angled; condylar width; condylar height; ramus height; total height (condylar height plus ramus height); and the presence or absence of the upper and lower first and second molars (Figure 1). 

All linear measurements were performed using a standardized stepwise protocol based on predefined anatomical landmarks.

Condylar height was measured as the linear distance from the most superior point of the condylar head to the deepest point of the sigmoid notch.

Condylar width was measured as the maximum horizontal dimension of the condylar head on the panoramic image.

Ramus height was measured from the deepest point of the sigmoid notch to the most inferior point of the mandibular angle.

Total mandibular height was calculated as the sum of condylar height and ramus height.

Intercondylar distance was defined as the linear distance between the most lateral points of the right and left condylar heads.

**Figure 2:**
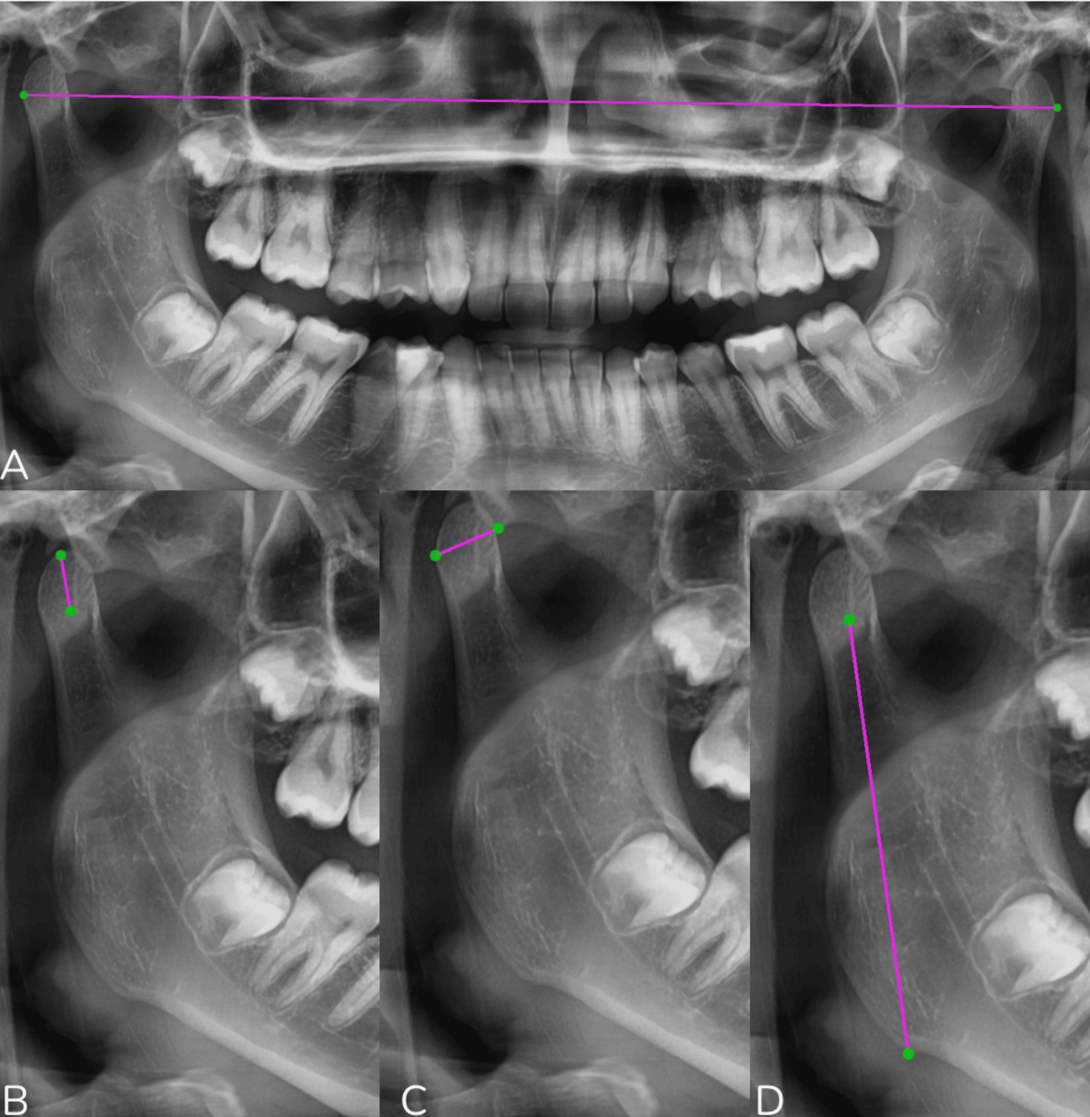
Panoramic radiographic measurements used in the study. (A) Measurement of intercondylar distance, defined as the linear distance between the most lateral points of the right and left mandibular condyles. (B) Condylar width, measured as the maximum horizontal dimension of the condylar head. (C) Condylar height, measured from the most superior point of the condylar head to the deepest point of the sigmoid notch. (D) Ramus height, measured from the deepest point of the sigmoid notch to the most inferior point of the mandibular angle.

The presence or absence of upper and lower first and second molars was also recorded for both sides of the mandible (Figure 2).

To assess measurement consistency, 50 randomly selected panoramic radiographs were re-evaluated after a four-week interval by the same examiner. In addition, the same subset of images was independently reviewed by a second author to verify landmark identification and measurement reproducibility. Intra-observer reliability for linear measurements was high, with intraclass correlation coefficients exceeding 0.85, while inter-observer agreement also demonstrated excellent consistency. Condylar morphology classification showed strong agreement between observers, indicating stable and reproducible categorical assessment.

Ethical considerations

Ethical approval for this research was granted by the Institutional Review Board (IRB) at the University of Hail. Informed consent was not required because the study used previously acquired panoramic radiographs and did not involve direct patient interaction. All images were de-identified following retrieval as part of routine clinical procedures to maintain patient privacy and confidentiality.

Statistical analysis

Descriptive and inferential statistical methods were employed for data analysis. The analysis was performed using SPSS, version 28 (IBM Corp., Armonk, NY). Descriptive statistics were used to calculate the frequency and distribution of condylar shapes, as well as the dental status. Chi-square tests or Fisher’s exact tests were used to assess associations between categorical variables, and a *P*-value of <0.05 was considered statistically significant. For all chi-square analyses, the chi-square statistics (χ²), degrees of freedom (df), and effect size (Cramer’s V) were considered when interpreting associations. Effect sizes were considered when interpreting statistically significant associations to avoid overreliance on *P*-values alone. Because measurements were derived from panoramic radiographs, analyses were interpreted at a population level rather than as precise morphometric estimates.

## Results

A total of 366 panoramic radiographs were analyzed in this study. Of these, 162 participants (44.3%) were males and 204 (55.7%) were females. The age distribution showed that 49 individuals (13.4%) were between 18-20 years old, 91 (24.9%) were 21-29 years old, 83 (22.7%) were 30-39 years old, 82 (22.4%) were 40-49 years old, 39 (10.7%) were 50-59 years old, and 22 participants (6%) were aged 60 years or older. Regarding the intercondylar distance, 22 individuals (6%) demonstrated values <150 mm, whereas the majority (240, 65.5%) fell within the 150-180 mm range, and 104 participants (28.4%) showed distances exceeding 180 mm. Examination of the right condylar shape revealed that the round morphology was the most prevalent, observed in 241 subjects (75.8%), followed by convex in 55 (15%), angled in 45 (12.3%), and flat in 25 (6.8%). Most participants had a right condylar width between 6 and 9 mm (265, 72.4%), while 49 (13.4%) measured <6 mm and 52 (14.2%) exceeded 9 mm. Right condylar height was <12 mm in 329 individuals (89.9%), 12-17 mm in 34 (9.3%), and >17 mm in only 3 subjects (0.8%). Right ramus height measurements showed that 136 individuals (37.2%) were <45 mm, 184 (50.3%) were between 45 and 55 mm, and 46 (12.6%) exceeded 55 mm. Similarly, the right total mandibular height was <60 mm in 262 participants (71.6%), 60-70 mm in 76 participants (20.8%), and >70 mm in 28 participants (7.7%). Evaluation of right posterior teeth revealed that the upper right first molar was present in 288 subjects (78.7%) and absent in 78 (21.3%). The upper right second molar was present in 306 individuals (83.6%) and absent in 60 (16.4%). The lower right first molar was present in 267 participants (73%) and absent in 99 (27%), whereas the lower right second molar was present in 298 individuals (81.4%) and absent in 68 (18.6%). On the left side, condylar shape assessment demonstrated that 262 subjects (71.6%) had a round morphology, 54 (14.8%) had a convex shape, 34 (9.3%) had an angled condyle, and 16 (4.4%) showed a flat configuration. Left condylar width measurements indicated that 31 individuals (8.5%) had widths <6 mm, 274 (74.9%) fell within the 6-9 mm range, and 61 (16.7%) had values >9 mm. The left condylar height was <12 mm in 329 participants (89.9%), between 12 and 17 mm in 37 individuals (10.1%), and no cases (0%) exceeded 17 mm. The left ramus height was <45 mm in 137 subjects (37.4%), between 45 and 55 mm in 182 (49.7%), and >55 mm in 47 subjects (12.8%). The left total height was <60 mm in 267 individuals (73%), between 60 and 70 mm in 68 (18.6%), and >70 mm in 31 individuals (8.5%). Evaluation of left posterior teeth showed that the upper left first molar was present in 289 participants (79%) and absent in 77 (21%). The upper left second molar demonstrated the same distribution, present in 289 (79%) and absent in 77 (21%). The lower left first molar was present in 257 subjects (70.2%) and absent in 109 (29.8%), while the lower left second molar was present in 284 participants (77.6%) and absent in 82 (22.4%) (Table 1).

**Table 1:** Distribution of demographic characteristics and radiographic morphometric categories.

Variable		*n* (%)
Gender		
Male		162 (44.3%)
Female		204 (55.7%)
Age groups		
18-20 years old		49 (13.4%)
21-29 years old		91 (24.9%)
30-39 years old		83 (22.7%)
40-49 years old		82 (22.4%)
50-59 years old		39 (10.7%)
60 years old or older		22 (6%)
Intercondylar distance		
<150 mm		22 (6%)
150-180 mm		240 (65.5%)
>180 mm		104 (28.4%)
Right condylar shape		
Round		241 (75.8%)
Angled		45 (12.3%)
Convex		55 (15%)
Flat		25 (6.8%)
Right condylar width		
<6 mm		49 (13.4%)
6-9 mm		265 (72.4%)
>9 mm		52 (14.2%)
Right condylar height		
<12 mm		329 (89.9%)
12-17 mm		34 (9.3%)
>17 mm		3 (0.8%)
Right ramus height		
<45 mm		136 (37.2%)
45-55 mm		184 (50.3%)
>55 mm		46 (12.6%)
Right total height		
<60 mm		262 (71.6%)
60-70 mm		76 (20.8%)
>70 mm		28 (7.7%)
Right molar teeth		
Upper right first molar	Present	288 (78.7%)
	Absent	78 (21.3%)
Upper right second molar	Present	306 (83.6%)
	Absent	60 (16.4%)
Lower right first molar	Present	267 (73%)
	Absent	99 (27%)
Lower right second molar	Present	298 (81.4%)
	Absent	68 (18.6%)
Left condylar shape		
Round		262 (71.6%)
Angled		34 (9.3%)
Convex		54 (14.8%)
Flat		16 (4.4%)
Left condylar width		
<6 mm		31 (8.5%)
6-9 mm		274 (74.9%)
>9 mm		61 (16.7%)
Left condylar height		
<12 mm		329 (89.9%)
12-17 mm		37 (10.1%)
>17 mm		0 (0%)
Left ramus height		
<45 mm		137 (37.4%)
45-55 mm		182 (49.7%)
>55 mm		47 (12.8%)
Left total height		
<60 mm		267 (73%)
60-70 mm		68 (18.6%)
>70 mm		31 (8.5%)
Left molar teeth		
Upper left first molar	Present	289 (79%)
	Absent	77 (21%)
Upper left second molar	Present	289 (79%)
	Absent	77 (21%)
Lower left first molar	Present	257 (70.2%)
	Absent	109 (29.8%)
Lower left second molar	Present	284 (77.6%)
	Absent	82 (22.4%)

Analysis of the association between intercondylar distance categories and demographic variables showed no statistically significant relationship with either gender or age. When comparing males and females (*P* = 0.630), the distribution across intercondylar distance groups was similar. Among males, 8 individuals (4.9%) had distances <150 mm, 105 (64.8%) were between 150 and 180 mm, and 49 (30.2%) measured >180 mm. Females demonstrated a comparable pattern, with 14 participants (6.9%) in the <150 mm category, 135 (66.2%) in the 150-180 mm category, and 55 (27%) with distances >180 mm, confirming the absence of a gender-based difference. Similarly, the comparison across six age groups revealed no statistically significant association (*P* = 0.340). In the 18-20-year group, 4 participants (8.2%) had distances <150 mm, 31 (63.3%) were between 150 and 180 mm, and 14 (28.6%) were >180 mm. In the 21-29-year group, 4 (4.4%) measured <150 mm, 63 (69.2%) were within 150-180 mm, and 24 (26.4%) exceeded 180 mm. For those aged 30-39 years, 7 (8.4%) were <150 mm, 52 (62.7%) were 150-180 mm, and 24 (28.9%) were >180 mm. In the 40-49-year group, 4 (4.9%) were <150 mm, 52 (63.4%) were 150-180 mm, and 26 (31.7%) measured >180 mm. Among the 50-59-year group, only 1 individual (2.6%) was <150 mm, 23 (59%) were in the 150-180 mm category, and 15 (38.5%) exceeded 180 mm. In the oldest group (≥60 years), 2 participants (9.1%) had distances <150 mm, 19 (86.4%) were 150-180 mm, and only 1 (4.5%) had distances >180 mm, reinforcing that age did not significantly influence intercondylar distance distribution (Table 2).

**Table 2:** Association of intercondylar distance categories with gender and age groups. Chi-square (χ²) tests were used to compare categorical variables across age and gender groups. df, degrees of freedom

Variables		Gender		Test statistics	Age groups						Test statistics
		Male	Female		18-20 years old	21-29 years old	30-39 years old	40-49 years old	50-59 years old	60 years old or older	
Intercondylar distance	<150 mm	8 (4.9%)	14 (6.9%)	χ² = 0.925, df = 2, *P*-value = 0.63, Cramer’s V = 0.05	4 (8.2%)	4 (4.4%)	7 (8.4%)	4 (4.9%)	1 (2.6%)	2 (9.1%)	χ² = 11.228, df = 10, *P*-value = 0.34, Cramer’s V = 0.124
	150-180 mm	105 (64.8%)	135 (66.2%)		31 (63.3%)	63 (69.2%)	52 (62.7%)	52 (63.4%)	23 (59%)	19 (86.4%)	
	>180 mm	49 (30.2%)	55 (27%)		14 (28.6%)	24 (26.4%)	24 (28.9%)	26 (31.7%)	15 (38.5%)	1 (4.5%)	

The analysis of right mandibular morphometric variables in relation to gender and age showed several significant and non-significant associations. For the right condylar shape, there was no statistically significant difference between males and females (*P* = 0.411). Among males, the round shape was observed in 114 individuals (70.4%), angled in 16 (9.9%), convex in 22 (13.6%), and flat in 10 (6.2%). Among females, the round shape occurred in 127 participants (62.3%), angled in 29 (14.2%), convex in 33 (16.2%), and flat in 15 (7.4%). In contrast, age groups showed a statistically significant association with right condylar shape (*P* = 0.024). In the 18-20-year group, 28 participants (56.1%) had a round condyle, while 9 (18.4%) were angled, 9 (18.4%) convex, and 3 (6.1%) flat. In the 21-29-year group, round, angled, convex, and flat shapes were found in 62 (68.1%), 14 (15.4%), 11 (12.1%), and 4 (4.4%) individuals, respectively. In the 30-39-year group, these distributions were 56 (67.5%), 4 (4.8%), 13 (15.7%), and 10 (12%), while the 40-49-year group showed 56 (68.4%) round, 6 (7.3%) angled, 17 (20.7%) convex, and 3 (3.7%) flat shapes. In the 50-59-year group, round, angled, convex, and flat shapes occurred in 28 (71.8%), 5 (12.8%), 2 (5.1%), and 4 (10.3%), respectively. Among participants aged 60 years or older, round condyles were present in 11 (50%), angled in 7 (31.8%), convex in 3 (13.6%), and flat in 1 (4.5%). The right condylar width demonstrated a statistically significant difference across gender (*P* = 0.049). In males, widths <6, 6-9, and >9 mm were observed in 22 (13.6%), 109 (67.3%), and 31 (19.1%) individuals, respectively. In females, the corresponding distributions were 27 (13.2%), 156 (76.5%), and 21 (10.3%). Age groups also showed a significant association (*P* = 0.008). Widths <6 mm ranged from 5 (10.2%) in the 18-20-year group to only 1 (4.5%) in the ≥60-year group. Widths 6-9 mm were most common across all ages, including 33 (67.3%) in the 18-20-year group and 16 (72.7%) in the ≥60-year group. Widths >9 mm peaked in the 50-59-year group at 12 individuals (30.8%). The right condylar height showed no statistically significant difference between genders (*P* = 0.729). Heights <12 mm were observed in 144 males (88.9%) and 185 females (90.7%), heights 12-17 mm in 17 males (10.5%) and 17 females (8.3%), and heights >17 mm in 1 male (0.6%) and 2 females (1%). However, age showed a highly significant association (*P* < 0.0001). Heights <12 mm ranged from 39 participants (79.6%) in the 18-20-year group to only 6 participants (27.3%) in the ≥60-year group. Heights 12-17 mm were especially frequent in the oldest group, with 16 individuals (72.7%), while heights >17 mm appeared only in very small numbers across younger groups. Right ramus height demonstrated a significant association with gender (*P* = 0.025). In males, heights <45, 45-55, and >55 mm occurred in 48 (29.6%), 93 (57.4%), and 21 (13%) participants, respectively. In females, these distributions were 88 (43.1%), 91 (44.6%), and 25 (12.3%). Age groups showed a highly significant difference (*P* < 0.0001). Heights >55 mm increased sharply with age, from 7 (14.3%) in the 18-20-year group to 16 (72.7%) in the ≥60-year group. The right total height showed no statistically significant gender difference (*P* = 0.051). Heights <60 mm were observed in 107 males (66%) and 155 females (76%), heights 60-70 mm in 43 males (26.5%) and 33 females (16.2%), and heights >70 mm in 12 males (7.4%) and 16 females (7.8%). A strong association with age was present (*P* < 0.0001). Heights >70 mm increased with age, from 3 individuals (6.1%) in the 18-20-year group to 16 individuals (72.7%) in the ≥60-year group. Regarding right molar presence, gender showed no significant association for any molar. The upper right first molar had *P* = 0.372, the upper right second molar had *P* = 0.394, the lower right first molar had *P* = 0.544, and the lower right second molar had *P* = 0.591. However, all molars demonstrated highly significant associations with age (all *P* < 0.0001). The presence of the upper right first molar decreased from 47 individuals (95.9%) in the 18-20-year group to only 11 (50%) in the ≥60-year group. A similar decline was seen in the upper right second molar, dropping from 48 (98%) in the youngest group to 15 (68.2%) in the oldest. The lower right first molar declined from 48 (98%) in the youngest group to 8 (36.4%) in the ≥60-year group, while the lower right second molar decreased from full presence in the youngest group (49; 100%) to 15 individuals (68.2%) in the ≥60-year group (Table 3).

**Table 3:** Relationship between demographics and right mandibular morphometric variables. Chi-square (χ²) tests were used to compare categorical variables across age and gender groups. *Statistically significant at *P*-value < 0.05. df, degrees of freedom

Variables		Gender		Test statistics	Age groups						Test statistics
		Male	Female		18-20 years old	21-29 years old	30-39 years old	40-49 years old	50-59 years old	60 years old or older	
Right condylar shape	Round	114 (70.4%)	127 (62.3%)	χ² = 2.875, df = 3, *P*-value = 0.411, Cramer’s V = 0.089	28 (56.1%)	62 (68.1%)	56 (67.5%)	56 (68.4%)	28 (71.8%)	11 (50%)	χ² = 27.659, df = 15, *P*-value = 0.024*, Cramer’s V = 0.159
	Angled	16 (9.9%)	29 (14.2%)		9 (18.4%)	14 (15.4%)	4 (4.8%)	6 (7.3%)	5 (12.8%)	7 (31.8%)	
	Convex	22 (13.6%)	33 (16.2%)		9 (18.4%)	11 (12.1%)	13 (15.7%)	17 (20.7%)	2 (5.1%)	3 (13.6%)	
	Flat	10 (6.2%)	15 (7.4%)		3 (6.1%)	4 (4.4%)	10 (12%)	3 (3.7%)	4 (10.3%)	1 (4.5%)	
Right condylar width	<6 mm	22 (13.6%)	27 (13.2%)	χ² = 6.029, df = 2, *P*-value = 0.049*, Cramer’s V = 0.128	5 (10.2%)	15 (16.5%)	14 (16.9%)	12 (14.6%)	2 (5.1%)	1 (4.5%)	χ² = 23.783, df = 10, *P*-value = 0.008*, Cramer’s V = 0.180
	6-9 mm	109 (67.3%)	156 (76.5%)		33 (67.3%)	67 (73.6%)	58 (69.9%)	66 (80.5%)	25 (64.1%)	16 (72.7%)	
	>9 mm	31 (19.1%)	21 (10.3%)		11 (22.4%)	9 (9.9%)	11 (13.3%)	4 (4.9%)	12 (30.8%)	5 (22.7%)	
Right condylar height	<12 mm	144 (88.9%)	185 (90.7%)	χ² = 0.631, df = 2, *P*-value = 0.729, Cramer’s V = 0.042	39 (79.6%)	90 (98.9%)	83 (100%)	80 (97.6%)	31 (79.5%)	6 (27.3%)	χ² = 142.524, df = 10, *P*-value < 0.001*, Cramer’s V = 0.441
	12-17 mm	17 (10.5%)	17 (8.3%)		8 (16.3%)	1 (1.1%)	0 (0%)	2 (2.4%)	7 (17.9%)	16 (72.7%)	
	>17 mm	1 (0.6%)	2 (1%)		2 (4.1%)	0 (0%)	0 (0%)	0 (0%)	1 (2.6%)	0 (0%)	
Right ramus height	<45 mm	48 (29.6%)	88 (43.1%)	χ² = 7.412, df = 2, *P*-value = 0.025*, Cramer’s V = 0.142	18 (36.7%)	38 (41.8%)	40 (48.2%)	22 (36.8%)	18 (46.2%)	0 (0%)	χ² = 92.169, df = 10, *P*-value < 0.001*, Cramer’s V = 0.355
	45-55 mm	93 (57.4%)	91 (44.6%)		24 (49%)	49 (53.8%)	39 (47%)	50 (61%)	16 (41%)	6 (27.3%)	
	>55 mm	21 (13%)	25 (12.3%)		7 (14.3%)	4 (4.4%)	4 (4.4%)	10 (12.2%)	5 (12.8%)	16 (72.7%)	
Right total height	<60 mm	107 (66%)	155 (76%)	χ² = 5.940, df = 2, *P*-value = 0.051, Cramer’s V = 0.127	35 (71.4%)	70 (76.9%)	71 (85.5%)	60 (73.2%)	25 (64.1%)	1 (4.5%)	χ² = 154.753, df = 10, *P*-value < 0.001*, Cramer’s V = 0.460
	60-70 mm	43 (26.5%)	33 (16.2%)		11 (22.4%)	21 (23.1%)	10 (12%)	19 (23.2%)	10 (25.6%)	5 (22.7%)	
	>70 mm	12 (7.4%)	16 (7.8%)		3 (6.1%)	0 (0%)	2 (2.4%)	3 (3.7%)	4 (10.3%)	16 (72.7%)	
Upper right first molar	Present	131 (80.9%)	157 (77%)	χ² = 0.820, df = 1, *P*-value = 0.365, Cramer’s V = 0.047	47 (95.9%)	81 (89%)	66 (79.5%)	62 (75.6%)	21 (53.8%)	11 (50%)	χ² = 40.104, df = 5, *P*-value < 0.001*, Cramer’s V = 0.331
	Absent	31 (19.1%)	47 (23%)		2 (4.1%)	10 (11%)	17 (20.5%)	20 (24.4%)	18 (46.2%)	11 (50%)	
Upper right second molar	Present	132 (81.5%)	174 (85.3%)	χ² = 0.958, df = 1, *P*-value = 0.328, Cramer’s V = 0.051	48 (98%)	85 (93.4%)	74 (89.2%)	55 (67.1%)	29 (74.4%)	15 (68.2%)	χ² = 38.213, df = 5, *P*-value < 0.001*, Cramer’s V = 0.323
	Absent	30 (18.5%)	30 (14.7%)		1 (2%)	6 (6.6%)	9 (10.8%)	27 (32.9%)	10 (25.6%)	7 (31.8%)	
Lower right first molar	Present	121 (74.7%)	146 (71.6%)	χ² = 0.446, df = 1, *P*-value = 0.504, Cramer’s V = 0.035	48 (98%)	78 (85.7%)	57 (68.7%)	51 (62.2%)	25 (64.1%)	8 (36.4%)	χ² = 45.091, df = 5, *P*-value < 0.001*, Cramer’s V = 0.351
	Absent	41 (25.3%)	58 (28.4%)		1 (2%)	13 (14.3%)	26 (31.3%)	31 (47.8%)	14 (35.9%)	14 (63.6%)	
Lower right second molar	Present	134 (82.7%)	164 (80.4%)	χ² = 0.322, df = 1, *P*-value = 0.570, Cramer’s V = 0.030	49 (100%)	84 (92.3%)	70 (84.3%)	57 (69.5%)	23 (59%)	15 (68.2%)	χ² = 42.004, df = 5, *P*-value < 0.001*, Cramer’s V = 0.339
	Absent	28 (17.3%)	40 (19.6%)		0 (0%)	7 (7.7%)	13 (15.7%)	25 (30.5%)	14 (41%)	7 (31.8%)	

The evaluation of left mandibular morphometric variables in relation to gender and age revealed multiple significant and non-significant associations. For the left condylar shape, there was no statistically significant difference between males and females (*P* = 0.479). Among males, the round shape was observed in 120 participants (74.1%), angled in 11 (6.8%), convex in 23 (14.2%), and flat in 8 (4.9%). Among females, the round shape was present in 142 individuals (69.6%), angled in 23 (11.3%), convex in 31 (15.2%), and flat in 8 (3.9%). Similarly, no statistically significant association was found between condylar shape and age (*P* = 0.175). In the 18-20-year group, round, angled, convex, and flat condyles occurred in 30 (61.2%), 9 (18.4%), 8 (16.3%), and 2 (4.1%) individuals, respectively. For those aged 21-29 years, these distributions were 69 (75.8%), 6 (6.6%), 12 (13.2%), and 4 (4.4%). In the 30-39-year group, 59 (71.1%) had round condyles, 4 (4.8%) angled, 16 (19.3%) convex, and 4 (4.8%) flat. In the 40-49-year group, round shapes appeared in 61 (74.4%), angled in 5 (6.1%), convex in 14 (17.1%), and flat in 2 (2.4%). The 50-59-year group showed 28 (71.8%) round, 5 (12.8%) angled, 3 (7.7%) convex, and 3 (7.7%) flat shapes, while the ≥60-year group showed 15 (68.2%) round, 5 (22.7%) angled, 1 (4.5%) convex, and 1 (4.5%) flat. Left condylar width showed no significant association with gender (*P* = 0.604). Widths <6, 6-9, and >9 mm were observed in 12 males (7.4%), 120 (74.1%), and 30 (18.5%), respectively, compared to 19 females (9.3%), 154 (75.5%), and 31 (15.2%). Age demonstrated no statistically significant association (*P* = 0.079). Widths <6 mm ranged from 5 individuals (10.2%) in the 18-20-year group to none (0%) in the ≥60-year group. Widths 6-9 mm were most frequent across all age categories, from 36 participants (73.5%) in the youngest group to 15 participants (68.2%) in the oldest. Widths >9 mm increased with age, reaching 7 individuals (31.8%) in the ≥60-year group. The left condylar height showed no significant gender difference (*P* = 0.386). Heights <12 mm were observed in 143 males (88.3%) and 186 females (91.2%), heights 12-17 mm in 19 males (11.7%) and 18 females (8.8%), and none exceeded 17 mm in either gender. Age, however, showed a highly significant association (*P* < 0.0001). Heights <12 mm occurred in 40 (81.6%) of those aged 18-20 years, 91 (100%) of those 21-29 years, 83 (100%) of those 30-39 years, 81 (98.8%) of those 40-49 years, 31 (79.5%) of those 50-59 years, but only 3 (13.6%) of those ≥60 years. Heights 12-17 mm were most common in the ≥60-year group with 19 individuals (86.4%), while present in only 9 (18.4%) individuals in the youngest group and absent in most middle age groups. No participants had heights >17 mm. The left ramus height showed a statistically significant gender association (*P* = 0.003). Heights <45 mm were reported in 45 males (27.8%) and 92 females (45.1%), heights 45-55 mm in 92 males (56.8%) and 90 females (44.1%), and heights >55 mm in 25 males (15.4%) and 22 females (10.8%). Age was highly significant (*P* < 0.0001). Heights <45 mm appeared in 21 (42.9%) of the 18-20-year group, 40 (44%) of the 21-29-year group, 36 (43.4%) of the 30-39-year group, but in none (0%) of those ≥60 years. Heights of 45-55 mm ranged from 21 individuals (42.9%) in the youngest group to 3 individuals (13.6%) in the oldest group. Heights >55 mm increased progressively with age, from 7 individuals (14.3%) in the 18-20-year group to 19 individuals (86.4%) in the ≥60-year group. The left total mandibular height showed no significant gender difference (*P* = 0.235). Heights <60 mm occurred in 111 males (68.5%) and 156 females (76.5%), heights of 60-70 mm in 35 males (21.6%) and 33 females (16.2%), and heights >70 mm in 16 males (9.9%) and 15 females (7.4%). However, age showed a highly significant association (*P* < 0.0001). Heights <60 mm were seen in 37 (75.5%) of those aged 18-20 years, 69 (75.8%) of those 21-29 years, and 70 (84.3%) of those 30-39 years, decreasing gradually with age until none (0%) of the ≥60-year group remained in this category. Heights >70 mm increased sharply with age, from 4 individuals (8.2%) in the youngest group to 17 individuals (77.3%) in the oldest. Regarding left molar presence, gender differences were mostly non-significant, except for the lower left first molar (*P* = 0.038). The upper left first molar was present in 129 males (79.6%) and 160 females (78.4%), and absent in 33 males (20.4%) and 44 females (21.6%) (*P* = 0.789). The upper left second molar was present in 127 males (78.4%) and 162 females (79.4%) (*P* = 0.897). The lower left first molar was present in 123 males (75.9%) and 134 females (65.7%), and absent in 39 males (24.1%) and 70 females (34.3%) (*P* = 0.038). The lower left second molar was present in 122 males (75.3%) and 162 females (79.4%), and absent in 40 males (24.7%) and 42 females (20.6%) (*P* = 0.378). Age demonstrated highly significant associations for all molars (all *P* < 0.0001). The presence of the upper left first molar declined from 46 individuals (93.9%) in the 18-20-year group to 9 individuals (40.9%) in the ≥60-year group. A similar decline was seen in the upper left second molar, decreasing from 48 (98%) to 13 (59.1%). The lower left first molar declined from 45 (91.8%) in the youngest group to 6 (27.3%) in the oldest, while the lower left second molar dropped from complete presence in the youngest group (49, 100%) to 11 individuals (50%) in the ≥60-year group (Table 4).

**Table 4:** Relationship between demographics and left mandibular morphometric variables. Chi-square (χ²) tests were used to compare categorical variables across age and gender groups. *Statistically significant at *P*-value < 0.05. df, degrees of freedom

Variables		Gender, *n* (%)		Test statistics	Age groups						Test statistics
		Male	Female		18-20 years old	21-29 years old	30-39 years old	40-49 years old	50-59 years old	60 years old or older	
Left condylar shape	Round	120 (74.1%)	142 (69.6%)	χ² = 2.481, df = 3, *P*-value = 0.479, Cramer’s V = 0.082	30 (61.2%)	69 (75.8%)	59 (71.1%)	61 (74.4%)	28 (71.8%)	15 (68.2%)	χ² = 19.924, df = 15, *P*-value = 0.175, Cramer’s V = 0.135
	Angled	11 (6.8%)	23 (11.3%)		9 (18.4%)	6 (6.6%)	4 (4.8%)	5 (6.1%)	5 (12.8%)	5 (22.7%)	
	Convex	23 (14.2%)	31 (15.2%)		8 (16.3%)	12 (13.2%)	16 (19.3%)	14 (17.1%)	3 (7.7%)	1 (4.5%)	
	Flat	8 (4.9%)	8 (3.9%)		2 (4.1%)	4 (4.4%)	4 (4.8%)	2 (2.4%)	3 (7.7%)	1 (4.5%)	
Left condylar width	<6 mm	12 (7.4%)	19 (9.3%)	χ² = 1.010, df = 2, *P*-value = 0.604, Cramer’s V = 0.053	5 (10.2%)	8 (8.8%)	11 (13.3%)	4 (4.9%)	3 (7.7%)	0 (0%)	χ² = 16.791, df = 10, *P*-value = 0.079, Cramer’s V = 0.151
	6-9 mm	120 (74.1%)	154 (75.5%)		36 (73.5%)	73 (80.2%)	57 (68.7%)	68 (82.9%)	25 (64.1%)	15 (68.2%)	
	>9 mm	30 (18.5%)	31 (15.2%)		8 (16.3%)	10 (11%)	15 (18.1%)	10 (12.2%)	11 (28.2%)	7 (31.8%)	
Left condylar height	<12 mm	143 (88.3%)	186 (91.2%)	χ² = 0.838, df = 1, *P*-value = 0.360, Cramer’s V = 0.048	40 (81.6%)	91 (100%)	83 (100%)	81 (98.8%)	31 (79.5%)	3 (13.6%)	χ² = 175.794, df = 5, *P*-value < 0.001*, Cramer’s V = 0.693
	12-17 mm	19 (11.7%)	18 (8.8%)		9 (18.4%)	0 (0%)	0 (0%)	1 (1.2%)	9 (20.5%)	19 (86.4%)	
	>17 mm	0 (0%)	0 (0%)		0 (0%)	0 (0%)	0 (0%)	0 (0%)	0 (0%)	0 (0%)	
Left ramus height	<45 mm	45 (27.8%)	92 (45.1%)	χ² = 11.672, df = 2, *P*-value = 0.003*, Cramer’s V = 0.179	21 (42.9%)	40 (44%)	36 (43.4%)	22 (26.8%)	18 (46.2%)	0 (0%)	χ² = 130.491, df = 10, *P*-value < 0.001*, Cramer’s V = 0.422
	45-55 mm	92 (56.8%)	90 (44.1%)		21 (42.9%)	47 (51.6%)	43 (51.8%)	54 (65.9%)	14 (35.9%)	3 (13.6%)	
	>55 mm	25 (15.4%)	22 (10.8%)		7 (14.3%)	4 (4.4%)	4 (4.8%)	6 (7.3%)	7 (17.9%)	19 (86.4%)	
Left total height	<60 mm	111 (68.5%)	156 (76.5%)	χ² = 2.894, df = 2, *P*-value = 0.235, Cramer’s V = 0.089	37 (75.5%)	69 (75.8%)	70 (84.3%)	65 (79.3%)	26 (66.7%)	0 (0%)	χ² = 160.125, df = 10, *P*-value < 0.001*, Cramer’s V = 0.468
	60-70 mm	35 (21.6%)	33 (16.2%)		8 (16.3%)	21 (23.1%)	12 (14.5%)	15 (18.3%)	7 (17.9%)	5 (22.7%)	
	>70 mm	16 (9.9%)	15 (7.4%)		4 (8.2%)	1 (1.1%)	1 (1.2%)	2 (2.4%)	6 (15.4%)	17 (77.3%)	
Upper left first molar	Present	129 (79.6%)	160 (78.4%)	χ² = 0.078, df = 1, *P*-value = 0.780, Cramer’s V = 0.015	46 (93.9%)	82 (90.1%)	64 (77.1%)	59 (72%)	29 (74.4%)	9 (40.9%)	χ² = 35.642, df = 5, *P*-value < 0.001*, Cramer’s V = 0.312
	Absent	33 (20.4%)	44 (21.6%)		3 (6.1%)	9 (9.9%)	19 (22.9%)	23 (28%)	10 (25.6%)	13 (59.1%)	
Upper left second molar	Present	127 (78.4%)	162 (79.4%)	χ² = 0.056, df = 1, *P*-value = 0.813, Cramer’s V = 0.012	48 (98%)	78 (85.7%)	63 (75.9%)	60 (73.2%)	27 (69.2%)	13 (59.1%)	χ² = 22.718, df = 5, *P*-value < 0.001*, Cramer’s V = 0.249
	Absent	35 (21.6%)	42 (20.6%)		1 (2%)	13 (14.3%)	20 (24.1%)	22 (26.8%)	12 (30.8%)	9 (40.9%)	
Lower left first molar	Present	123 (75.9%)	134 (65.7%)	χ² = 4.527, df = 1, *P*-value = 0.033*, Cramer’s V = 0.111	45 (91.8%)	75 (82.4%)	59 (71.1%)	47 (57.3%)	25 (64.1%)	6 (27.3%)	χ² = 44.083, df = 5, *P*-value < 0.001*, Cramer’s V = 0.347
	Absent	39 (24.1%)	70 (34.3%)		4 (8.2%)	16 (17.6%)	24 (28.9%)	35 (42.7%)	14 (35.9%)	16 (72.7%)	
Lower left second molar	Present	122 (75.3%)	162 (79.4%)	χ² = 0.874, df = 1, *P*-value = 0.378, Cramer’s V = 0.049	49 (100%)	84 (92.3%)	67 (80.7%)	49 (59.8%)	24 (61.5%)	11 (50%)	χ² = 56.376, df = 5, *P*-value < 0.001*, Cramer’s V = 0.392
	Absent	40 (24.7%)	42 (20.6%)		0 (0%)	7 (7.7%)	16 (19.3	33 (40.2%)	15 (38.5%)	11 (50%)	

## Discussion

A range of two-dimensional (2D) and three-dimensional (3D) imaging modalities can be used to assess mandibular condyle and ramus morphology [12-20]. Although cone-beam computed tomography (CBCT) offers superior diagnostic detail and is considered the gold standard [12,13], its higher radiation dose and cost limit its routine use [20]. Panoramic radiographs, on the other hand, are low-cost, widely available, and involve significantly less radiation [21]. Previous studies have shown that when patient positioning is optimized, vertical and morphometric measurements on panoramic radiographs are sufficiently reliable for clinical and research purposes [18,22]. Condylar and ramal heights have also been effectively used in 2D evaluations to assess asymmetry and skeletal pattern differences [15-17,21,23]. In this study, high-quality panoramic radiographs with standardized head positioning were used, providing an efficient and practical method for evaluating condylar morphology and dimensions in a study population.

Recent epidemiological evidence has demonstrated a notable prevalence of temporomandibular disorders in young adult populations, emphasizing that assessment of mandibular and occlusal relationships should be interpreted within an appropriate clinical context [4].

A CBCT-based study in a Turkish adult population demonstrated clear sexual dimorphism in several mandibular parameters, including condylar length, coronoid length, mandibular length, minimal ramus breadth, and transverse measurements such as bigonial and bicondylar widths, while mandibular angle did not differ significantly between sexes [24]. In comparison, our digital panoramic radiography (DPR)-based study identified ramus height as the most distinctly sexually dimorphic parameter, showing significant differences between males and females, whereas condylar height and most condylar width measurements did not differ significantly, likely reflecting both population differences and the lower dimensional sensitivity of 2D imaging.

A recent meta-analysis similarly concluded that males typically exhibit larger vertical and transverse mandibular dimensions [25] and highlighted vertical ramus height as one of the most reliable indicators for sex estimation. Our findings align with this, as ramus height was the only measurement with consistent sexual dimorphism in our sample, while condylar height and width showed no significant sex-based differences.

In this study, the round-shaped condyle was the most common morphology on both sides (75.8% on the right and 71.6% on the left), followed by the convex shape, then the angled shape, while the flat morphology was the least frequent. These findings are consistent with previous studies by Oliveira-Santos et al. and Sahithi et al., who likewise reported the round/oval configuration as the predominant condylar form in their samples [26,27]. However, our study differed in the ranking of secondary morphologies. Whereas those studies identified the angled condyle as the second most common shape, our population demonstrated a higher prevalence of the convex morphology.

Overall, the findings of this study reinforce the clinical value of panoramic radiography as a practical and efficient tool for evaluating condylar morphology and vertical mandibular dimensions in large cohorts. Although panoramic radiographs cannot replace CBCT for detailed morphometric assessment, their low radiation exposure and accessibility make them suitable for baseline screening and epidemiological research. The observed sexual dimorphism in ramus height and the strong age-related trends in mandibular measurements highlight the importance of considering demographic factors when interpreting craniofacial radiographs.

Limitations

This study has several limitations. The use of 2D panoramic radiographs introduces magnification and projection errors that limit precise morphometric assessment and prevent evaluation of mediolateral or volumetric condylar dimensions. The retrospective cross-sectional design restricts causal interpretation, and age-related differences may reflect cohort effects rather than true remodeling. Although intra-observer reliability was assessed, inter-observer reliability and key functional or skeletal factors, such as facial type, occlusion, and parafunctional habits, were not evaluated. Future multi-center studies incorporating CBCT imaging and broader clinical parameters are recommended to strengthen these findings.

## Conclusions

This study demonstrated that the round/oval condylar morphology is the predominant form in adults in Hail, Saudi Arabia. Among linear measurements, ramus height showed the most consistent sexual dimorphism, whereas condylar height and width did not differ significantly between males and females. Strong age-related trends were observed in vertical mandibular dimensions and posterior dentition status. These findings suggest that panoramic radiography may be useful for population-level screening and epidemiological assessment of condylar morphology and basic mandibular metrics. However, they should be interpreted cautiously and considered hypothesis-generating rather than definitive. CBCT remains necessary for detailed three-dimensional evaluation when clinically indicated.
